# The Placenta in Gestational Diabetes: An Integrated Review on Metabolic Pathways, Genetic, Epigenetic and Ultrasound Biomarkers for Clinical Perspectives

**DOI:** 10.3390/ijms27020919

**Published:** 2026-01-16

**Authors:** Giovanni Tossetta, Roberto Campagna, Arianna Vignini, Giuseppe Maria Maruotti, Mariarosaria Motta, Chiara Murolo, Laura Sarno, Camilla Grelloni, Monia Cecati, Stefano Raffaele Giannubilo, Andrea Ciavattini

**Affiliations:** 1 Department for the Promotion of Human Science and Quality of Life, San Raffaele Roma University, Via di Val Cannuta, 247, 00166 Rome, Italy; 2 Department of Clinical Sciences, Section of Biochemistry, Biology and Physics, Università Politecnica Delle Marche, 60126 Ancona, Italy; 3 Gynecology and Obstetrics Unit, Department of Public Health, University of Naples Federico II, 80131 Naples, Italy; 4 Department of Clinical Sciences, Clinic of Obstetrics and Gynaecology, Università Politecnica Delle Marche, 60123 Ancona, Italy

**Keywords:** gestational diabetes mellitus, genetic factors, epigenetic modifications, dysregulated metabolic pathways, serum biomarkers, sonographic markers

## Abstract

Pregnancies complicated by diabetes, including pregestational and gestational diabetes mellitus, are associated with increased maternal and fetal morbidity. Early identification of at-risk pregnancies is crucial for timely intervention and improved outcomes. Emerging evidence highlights the interplay of genetic predisposition, epigenetic modifications, and non-invasive biomarkers in the early detection of diabetic pregnancies. Genetic factors influencing insulin signaling, glucose metabolism, and pancreatic β-cell function may contribute to susceptibility to gestational hyperglycemia. Concurrently, epigenetic alterations, such as DNA methylation and histone modifications in maternal and placental tissues, have been linked to dysregulated metabolic pathways and adverse pregnancy outcomes. Non-invasive biomarkers, including circulating cell-free DNA and microRNAs in maternal blood, show promise for early diagnosis by offering a safer and more practical alternative to invasive testing. Integrating genetic, epigenetic, and molecular marker data could enhance risk stratification and enable personalized monitoring and management strategies. This review synthesizes current knowledge on the molecular underpinnings of diabetic pregnancies, evaluates the potential of emerging biomarkers for early diagnosis, and discusses the challenges and future perspectives for translating these findings into clinical practice. Understanding these mechanisms may pave the way for precision medicine approaches, ultimately improving maternal and neonatal outcomes in pregnancies affected by diabetes.

## 1. Introduction

Gestational diabetes mellitus (GDM) is one of the most common metabolic disorders during pregnancy, characterized by glucose intolerance that occurs during gestation with various degrees of severity.

The overall incidence of GDM ranges from 5% to 15%, and this value is rising due to the spread of obesity and the tendency for women to become pregnant at an older age [1,2].

GDM is an insidious condition that places pregnant women at higher risk of perinatal complications and macrosomia (infants born with excessive birth weight), as well as increasing the likelihood of developing type 2 diabetes mellitus (T2DM) in both mother and child [3,4,5].

The placenta is a key organ for the development of mammalian life and is responsible for transporting essential substances and waste products between the mother and the fetus.

In addition to these exchange functions, placental tissue produces and secretes a wide range of hormones and cytokines, which are critical mediators of maternal adaptation to pregnancy, and manages fetal maturation and oxygenation during labour and delivery [6,7].

Several factors regulate placental transport, including uteroplacental and umbilical blood flow, the surface area available for exchange, placental metabolic activity, and the expression and function of specific transport proteins within the placental barrier.

In general, transplacental transfer of substances is mediated by passive diffusion, active transport and facilitated diffusion, as well as phagocytosis and pinocytosis.

Among these, passive diffusion (i.e., the permeation of substances across the concentration gradient) is the predominant form of transplacental transport.

There are also active transporters at the level of syncytiotrophoblasts (maternal and fetal parts) whose regulation depends largely on hormonal action.

The microvilli of syncytiotrophoblasts express numerous hormone receptors, including receptors for insulin, IGF-I and leptin [8,9].

Its strategic position between the maternal and fetal bloodstreams makes the placenta not only a regulator of fetal nutrition but also a target of maternal metabolic changes associated with pregnancy, including GDM. Specifically, GDM can foster abnormalities in both placental development and function.

On gross examination, placentas from pregnancies complicated by GDM show a significantly higher weight and a reduced fetoplacental weight ratio (F:P), compared to those from physiological pregnancies.

These changes could be caused by an increase in the number of cells on the one hand and an excess of water content on the other.

The most significant changes occur at the microscopic level; during the second half of pregnancy, GDM is associated with impaired villous maturation and altered placental vascularization [10,11]. In placentas from pregnancies complicated by GDM, there are no abnormalities in size, number of terminal villi, or total villous exchange surface area, but a direct correlation has been demonstrated between mean glycemic control and villous vessel area [12].

More specifically, some authors have observed fibrinoid necrosis of chorionic villi and villous oedema in the placentas of women with GDM. This finding could be linked to placental angiogenesis mechanisms, which are regulated by various factors including VEGF (vascular endothelial growth factor), FGF-2 (fibroblast growth factor) and PPAR-γ (peroxisome proliferator-activated receptor) [13].

In fact, the literature provides evidence of increased expression of FGF-2 mRNA and reduced regulation of its receptor FGF-2R and PPAR-γ in the placentas of pregnancies with GDM [14,15].

GDM has also been associated with a reduction in adhesion and junction proteins and therefore with impaired placental barrier function [16].

Another fundamental role of the placenta is the modulation of the effects of GDM on both the mother and the fetus, through the regulation of nutrient and metabolite transfer between them and the response to maternal metabolic signals.

Placental disfunctions caused by maternal hyperglycemia can influence fetal growth, endothelial function, and the child’s long-term metabolic profile [17].

Furthermore, placental oxidative stress triggered by a high-glucose environment can interfere with enzyme activity [18,19] and reduce the expression of genes encoding proteins necessary for physiological fetal growth [20,21,22]. Animal studies have shown that this effect can be mitigated by administering certain adipokines, such as chemerin, which help to reduce excessive fetal growth [23].

Emphasis on studies providing “clinical insights and mechanistic understanding” of GDM placental pathways ensured relevance to the review’s translational objectives, avoiding purely descriptive papers. Unlike prior reviews that focus primarily on epigenetics alone (e.g., DNA methylation changes in placenta) or genetics in isolation, this manuscript explicitly traces the causal chain: genetic predisposition—epigenetic reprogramming—metabolic pathway dysregulation—oxidative/inflammatory stress—placental dysfunction. Most placental reviews emphasize post-delivery tissue analysis, but this work prioritizes translational biomarkers from maternal blood (cfDNA, exosomal circRNAs, miR-29a/miR-223 panels) and ultrasound (3D-PD vascular indices) for first-trimester screening. It critically evaluates their diagnostic potential against the OGTT gold standard, including contradictory results. Unlike descriptive reviews, this manuscript includes forward-looking sections on epigenetic therapies (HDAC inhibitors), AI-driven risk models, and transgenerational DOHaD studies, grounded in recent consensus guidelines. It emphasizes multi-ancestry validation and reversibility testing (diet-methyl donor effects), addressing gaps in ethnic diversity highlighted in prior critiques [24,25].

## 2. Methods

To identify relevant studies, we conducted a comprehensive literature search across several databases, including PubMed, Scopus, and Web of Science. The search aimed to locate significant English-language research articles and reviews published in peer-reviewed journals over the past two decades (2000–2024); studies published before this period were considered only if deemed foundational. Foundational studies on physiological and diabetic-complicated pregnancies published before this period were also included to provide historical context and support mechanistic interpretations. Our review focused on both in vivo human and animal studies to enhance translational relevance. Studies were excluded if they lacked significant outcomes related to gestational diabetes-associated metabolic alterations.

The search terms were carefully selected to encompass a wide range of research on the relationship between GDM and deregulated metabolic pathways or predisposing genetic factors. The following keywords and phrases, in various combinations, were used: ‘genetic predisposing factors’, ‘healthy pregnancies’, ‘diabetic pregnancies’, ‘deregulated metabolic pathways’, ‘epigenetic modifications’, ‘sonographic markers’, ‘serum markers’, and ‘early diagnosis’. Boolean operators (AND, OR, NOT) were applied to refine and expand the search.

Inclusion criteria focused on studies exploring the consequences of deregulated metabolic pathways on successful pregnancies, as well as research on non-invasive biomarkers for the early diagnosis of GDM. Exclusion criteria ruled out non-English studies, articles published before 1994, and those lacking relevant clinical or experimental data. This approach ensured a thorough and wide-reaching review, emphasizing studies that provided clinical insights and a mechanistic understanding of the relationship between deregulated metabolic pathways, epigenetic factors, and pregnancies complicated by diabetes.

## 3. Predisposing Factors for GDM

Predisposing factors for GDM include both inherited genetic susceptibility and environmentally driven epigenetic modifications, which jointly contribute to altered metabolic adaptation during pregnancy.

### 3.1. Genetic Factors

Genetic factors are relevant components in assessing the risk of GDM, as they often interact with other environmental and behavioral factors.

Dysregulation of glucose and lipid metabolism, insulin signaling, and inflammatory pathways plays a central role in the pathogenesis of GDM, and several GWAS-identified genetic variants are implicated in these mechanisms. Transcription factor 7-like 2 (TCF7L2), melatonin receptor 1B (MTNR1B), and glucokinase (GCK) are key genes involved in β-cell function and glucose tolerance during gestation and are among the most extensively studied genetic determinants of GDM risk [1].

Several authors have shown that genetic mutations in the *TCF7L2* gene are associated with reduced insulin secretion during hyperglycemic conditions [26,27] while alterations in *MTNR1B* could affect insulin secretion and predispose the individual to higher fasting hyperglycemia [28]. Furthermore, individuals carrying *GCK* variants may suffer from more pronounced postprandial glycemic spikes [29].

Zhen et al. conducted a GWAS study focusing on the genetic and molecular basis of GDM in over 30,000 pregnant women in China.

In relation to blood glucose regulation, the authors identified four susceptibility loci on the genome (*MTNR1B*, *CDKAL1*, *SLC30A8*, *CPO*) that could be functionally linked to GDM [30].

The coexistence of multiple genetic polymorphisms and adverse environmental factors (such as obesity, physical inactivity, and unhealthy dietary patterns) has been associated with an increased risk of GDM.

Single nucleotide polymorphisms (SNPs) in *TCF7L2*, *MTNR1B*, *CDKAL1*, *GCK*, and *SLC30A8* that have been associated with beta-cell dysfunction and impaired glucose tolerance in pregnancy seem to be of particular interest [31,32].

For instance, the “G” allele of SNP rs10830963 within the *MTNR1B* gene has been linked to an increased risk of GDM, with an odds ratio of up to 1.84, confirming its relevance in different populations [32].

In addition, in a systematic review, Zhang et al. highlighted a link between the risk of GDM and nine SNPs in seven different genes, including *TCF7L2*, *MTNR1B*, and *CDKAL1* [33].

A growing body of literature has recently focused on genetic risk scores (GRS) that combine different SNPs, showing that carrying multiple risk variants can increase the risk of developing GDM. This approach could enable the identification of women at high risk of GDM in the pre-conception period in the future, paving the way for targeted prevention strategies [34,35].

William et al. built a predictive model of genetic risk for developing T2DM after pregnancy in women who experienced pregnancy complicated by GDM. The authors concluded that genetic profiling of patients could help identify women at higher risk of developing long-term metabolic complications [36].

In a randomized controlled trial on 2418 pregnant women screened for GDM, Ramos-Levi et al. found a cluster of SNPs conferring a higher risk of developing GDM. On the other hand, the authors also demonstrated that a Mediterranean diet could offset the effect of these risk variants and thus reduce the risk of developing GDM [37].

An analysis of the literature also highlights several less extensively studied SNPs that may contribute to GDM susceptibility. For example, polymorphisms in the *HTR2B* gene (rs17619600) and in the Lin28/let-7 cluster have been related to postprandial glucose levels and a higher risk of GDM [38,39]. Furthermore, the rs2237892 polymorphism of the *KCNQ1* gene has been associated with an increased risk of GDM and elevated blood glucose levels during pregnancy, as demonstrated by a study that included both a case–control analysis and a meta-analysis conducted on Asian populations. This evidence suggests that genetic factors may influence not only the onset of GDM but also the severity of its metabolic consequences [40].

When analyzing personal risk factors, many studies have found that GDM is more common in women with a family history of T2DM or GDM. A prospective study of 1129 pregnant women found that first-degree relatives with T2DM increased the risk of developing GDM by approximately twofold (OR 1.91; 95% CI 1.16–3.16). If both first-degree and second-degree relatives were affected, the risk was even higher, with an odds ratio of 2.64 (95% CI 1.41–4.94) [41].

This hypothesis was also supported by a study by Lewandowska, which showed that having a parent with diabetes (e.g., mother or father) increases the risk of GDM three to four times [42]. The fact that a positive family history of diabetes significantly increases an individual’s risk of GDM suggests that there may be a common genetic background between T2DM and GDM. Research on gene associations and polygenic risk scores indicates that, in many cases, genetic variants associated with T2DM are also linked to GDM, reinforcing the hypothesis of shared pathophysiological mechanisms between the two conditions [33].

Indeed, an interesting trans-ancestral GWAS meta-analysis confirmed that some loci related to GDM risk, including *TCF7L2* and *MTNR1B*, are already known to be associated with T2DM as well, supporting genetic overlap between these two conditions [43]. This suggests that GDM risk is conferred not only by individual common variants with a relatively large effect, but also by complex inheritance patterns of combinations of variants that affect pancreatic β-cell function, insulin sensitivity, and overall metabolic control.

A GRS based on common variants for T2DM risk was also found to be useful for predicting GDM risk, with each additional risk allele increasing the cumulative risk of GDM by 10% [44]. Finally, evidence indicates that, in addition to a family history of diabetes, obesity (as reflected by increased body mass index (BMI)) interacts with genetic susceptibility to further increase GDM risk [42]. Although multiple genetic variants have been associated with GDM risk, most effect sizes are modest and often population-specific, limiting their standalone predictive value. Furthermore, limited replication across cohorts and ethnic groups underscores the need for larger, multi-ethnic studies before clinical translation. The main genetic variants and genetic risk factors associated with GDM, including GWAS-identified loci, candidate gene polymorphisms, and polygenic risk scores, are summarized in Table 1.

### 3.2. Epigenetic Factors

Beyond inherited genetic susceptibility, epigenetic mechanisms represent a dynamic interface between maternal metabolic environment and placental gene regulation.

Epigenetics is a key mechanism through which the intrauterine environment regulates placental gene expression without altering the DNA sequence. In response to maternal environmental conditions, the placenta can modulate metabolic and endocrine pathways but also induce epigenetic modifications. These modifications (e.g., changes in imprinting, DNA methylation, and microRNA regulation) can have important consequences on the functioning of fetal-maternal exchanges, fetal growth, and long-term metabolic programming [45,46,47].

A growing body of evidence has shown that placentas of pregnant women with hyperglycemia undergo both global and specific changes in DNA methylation. For example, a study of over 1000 placentas found that mothers with GDM had significantly higher levels of global placental DNA methylation than normoglycemic pregnant women (3.22 ± 0.63% vs. 3.00 ± 0.46%; *p* = 0.013). Moreover, a strong association was found between higher methylation levels and the presence of GDM, even after adjusting for maternal age and BMI [48].

A further epigenome-wide association study (EWAS) of 42 patients showed that the placentas of women with GDM had 12,210 differentially methylated CpGs (DMCs) that mapped to 3875 genes. These epigenetically altered genes were enriched in the Wnt and cadherin signaling pathways, both of which are critical in placentation and embryogenesis [49]. Recent studies have identified specific methylated DNA regions where genes such as *IGF2*, *PPARγ*, and *LEP* are located, which regulate cell proliferation, trophoblast function, and metabolic reactivity of the placenta [49,50].

Recent findings suggest that altered DNA methylation at CpG sites may affect gene expression. A study integrating methylome and transcriptome data showed that 155 genes with alterations in both methylation and expression are present in the placental tissue of pregnant women with GDM. These altered genes were enriched in pathways related to “insulin signaling,” “insulin secretion,” and T2DM [51].

In addition to global methylation, specific genes may also exhibit altered methylation levels in the placenta of women with GDM. Chen and colleagues identified increased promoter methylation of the TRIM67 gene in the placentas of mothers who had GDM. Hypermethylation of the TRIM67 promoter region showed a significant positive correlation with 1- and 2 h oral glucose tolerance test (OGTT) values and a negative correlation with blood lipoprotein levels [52]. The review by Dalfrà et al. gathered evidence that reinforced the link between maternal glycemic status and placenta-specific epigenetic reprogramming [53].

A longitudinal study conducted by Linares-Pineda et al. also showed that these epigenetic modifications can have transgenerational effects, as mothers with GDM have offspring with increased metabolic susceptibility [54]. Furthermore, the methylation of key genes such as *PPARγ* and *IGF2* can be influenced by maternal diet and exposure to endocrine-disrupting substances, with consequences for fetal growth [55].

Maternal nutrition can also influence the epigenetic profile of the placenta: a recent paper evaluated the contribution of maternal diet, including nutrients such as methyl group donors (e.g., betaine, folate, and choline) on DNA methylation in placental tissue and cord blood. The data showed that the level of betaine in the maternal diet was negatively correlated with *IGF2* expression in the placenta, suggesting the existence of a mechanism through which maternal diet can modulate the placental epigenetic profile and, consequently, regulate fetal growth [56].

Conditions such as GDM can alter the epigenetic programming in the placenta and embryo, which may lead to an increased risk of obesity, T2DM, and metabolic dysfunction in the future life of the child [57]. A recent review highlighted that epigenetic modifications (e.g., DNA methylation, miRNA, lncRNA, histone modifications) are recurrent in the placentas of pregnancies complicated by GDM and can alter fetal-placental endothelial function, vascular permeability, and metabolic homeostasis [58].

Despite growing evidence, several questions remain unresolved. It is still unclear whether the epigenetic alterations observed in the placenta of pregnancies complicated by GDM are a consequence of maternal hyperglycemia or result from other conditions such as obesity, inflammation, or changes in the placental microenvironment. To answer this question, Meyrueix et al. enrolled obese and non-obese mothers, comparing placentas unaffected by GDM with those affected by GDM, and the results suggested that these epigenetic changes are specifically associated with GDM [49].

Finally, Linares-Pineda et al. highlighted how an epigenetic risk score for T2DM can also be predictive for GDM. This result supports the hypothesis that T2DM and GDM are closely linked by molecular, genetic, and even epigenetic linkage [59].

Although several genes with differential methylation have been identified, only a limited number of studies have succeeded in linking these genes to obstetric outcomes, including fetal birth weight, placental vascularization, or postnatal metabolic status. For example, although Wang and his team highlighted changes in methylation in 30 placentas, no significant correlations with metabolic biomarkers in cord blood were observed [60]. Epigenetic findings provide valuable insight into gene–environment interactions; however, their interpretation is challenged by tissue specificity, temporal variability during pregnancy, and limited longitudinal validation. These factors currently constrain the use of epigenetic markers as independent diagnostic tools. A comprehensive overview of epigenetic alterations associated with GDM, including DNA methylation changes, miRNA dysregulation, and epigenetic risk scores, is provided in Table 2.

Together, genetic predisposition and epigenetic reprogramming establish a molecular background that predisposes the placenta to dysregulated metabolic signaling under conditions of maternal hyperglycemia.

## 4. Dysregulated Metabolic Pathways in Diabetic Placenta

Placental metabolic pathway disturbances in GDM arise from the interaction between genetic susceptibility and epigenetically mediated alterations in gene expression, ultimately resulting in impaired nutrient sensing and transport. The placenta is a transient organ that transports and regulates the metabolism of glucose, lipids, and amino acids for the intrauterine development of the fetus [61]. Placental balance, trophoblast activity, and fetal metabolic programming are affected by complex disruptions of metabolic pathways driven by elevated maternal glucose and insulin levels in GDM [62]. The main metabolic pathways that are dysregulated in GDM are discussed in the following sections, which also highlight the implications of molecular mechanisms for fetal development.

### 4.1. Glucose Metabolism

Glucose, the main source of energy for the fetus during pregnancy, crosses the placenta mainly through GLUT1 and GLUT3 transporters [63].

The number of these transporters increases progressively during physiological pregnancy to meet the growing metabolic needs of the fetus [64]. In GDM, increased expression of GLUT1 and GLUT3 transporters enhances the amount of glucose available to the fetus. According to research conducted by Aldahmash and colleagues, who analyzed 65 placentas from both groups [65], GLUT1 and GLUT3 levels were significantly higher in the placentas of women with GDM than in those of healthy pregnant women.

Consequently, high glucose levels in the fetal environment cause activate glycolytic pathways, leading to accumulation of metabolites such as fructose-6-phosphate and glucose-6-phosphate. The presence of these two molecules stimulates further metabolic pathways, including the hexosamine biosynthesis pathway, which is necessary to produce uridine diphosphate-N-acetylglucosamine (UDP-GlcNAc). UDP-GlcNAc is important for both N-linked and O-linked glycosylation, which are forms of modification that occur during or after protein synthesis and play a crucial role in regulating protein function and expression [66].

Through a process called O-GlcNAcylation (O-GlcNAc), UDP-GlcNAc performs a post-translational modification of proteins [67]. O-GlcNAc is particularly significant in placental tissue as it regulates the activation of several nuclear transcription factors [68]. The nuclear factor kappa-light-chain-enhancer of activated B cells (NF-κB) is altered by O-GlcNAc in the placentas of rats under conditions of high blood sugar. This causes the factor to migrate to the nucleus, which increases the synthesis of pro-inflammatory cytokines [69]. Hypoxia-inducible factor-1alpha (HIF-1α), a crucial regulator of genes essential for placental vascular development, has been found to decrease in animal experiments when O-GlcNAc levels decrease [70]. O-GlcNAc has also been identified as a novel regulator of ChREBP, an essential transcription factor that stimulates the expression of genes involved in hepatic glycolysis and fat synthesis in response to glucose levels [71].

Advanced glycation end products (AGEs) are also produced under conditions of high blood sugar. In a recent study conducted by Zhang et al., 126 pregnant women with GDM and 85 pregnant women of comparable age without GDM were included as controls. The results revealed that, compared to the control group, the GDM group had significantly higher serum levels of AGEs and malondialdehyde, which are markers of oxidative stress and lipid peroxidation [72]. Excess lactate is produced in placentas associated with GDM due to insulin resistance and elevated blood sugar levels. According to a metabolomics study by Jansson et al., prenatal macrosomia and neonatal hyperinsulinemia are linked to the accumulation of glucose and lactate in diabetic placentas [73]. In addition to causing abnormal nutrient transport, altered glucose metabolism in the maternal environment also modifies villi structure and function, affects placental blood flow, and produces long-term epigenetic changes [51,74,75]. Elevated glucose levels in GDM-affected placentas cause disruption of mTOR/AMPK pathways, which alters trophoblast development and protein synthesis.

### 4.2. Energy Metabolism and Signaling Pathways

AMPK and mTOR act as sensors for the cell’s energy balance, and represent the system that regulates the homeostasis of placental cells [76,77]. The heterotrimeric AMPK protein, composed of three distinct subunits (α, β, and γ), is essential for the activation of catabolic functions in the cell. To activate AMPK, AMP must bind to the γ subunit, and certain threonine residues on the α subunit must be phosphorylated [78]. To restore energy balance in the placental cell, catabolic activities such as glycolysis, β-oxidation of fatty acids, and autophagy are stimulated when AMPK is activated with decreasing ATP levels and increasing AMP/ATP ratio [79]. Unlike AMPK, mTOR has the opposite purpose. In response to insulin, growth hormones, and food availability, it promotes protein synthesis, lipogenesis, and cell proliferation, driving anabolic processes [80].

Together, AMPK and mTOR maintain a dynamic balance during healthy pregnancies that meets the energy needs of the placenta without compromising fetal development or cellular homeostasis [81]. However, maternal hyperglycemia and hyperinsulinemia cause continuous activation of mTOR in pregnancies complicated by GDM, which in turn causes a decrease in AMPK activity [82,83].

This imbalance results in dysregulation of placental cellular homeostasis: long-term activation of mTOR leads to excessive protein synthesis and lipid peroxidation, while autophagic activity is reduced, resulting in the accumulation of toxic protein aggregates and increased production of reactive oxygen species (ROS) [79,82]. Increased expression of protein transporters such as GLUT (glucose transporter) and FATP (fatty acid transport protein) is associated with persistent mTOR activation. Conditions such as macrosomia and neonatal hyperinsulinemia may result from this increase in maternal nutrient supply to the fetus [80,84]. The decrease in mitochondrial biogenesis and oxidative capacity is linked to altered mTOR and AMPK activity in pregnancies affected by GDM. It has been shown that two important regulators of mitochondrial biogenesis, PGC-1α (peroxisome proliferator-activated receptor gamma coactivator 1-alpha) and NRF1 (nuclear respiratory factor 1), decrease in level, causing a malfunction of the electron transport chain that reduces ATP production [85,86]. The accumulation of ROS can therefore damage cells and trigger inflammatory processes, which can affect placental function and embryonic development [87]. Maternal hyperglycemia and hyperinsulinemia in patients with GDM can alter the distribution and activity of villous capillaries, affect placental blood flow and promote oxidative stress and placental hypoxic conditions [88].

### 4.3. Lipid and Amino Acidic Metabolism

During the anabolic phase of the first trimester of a typical pregnancy, the maternal body begins to accumulate lipids in adipose tissue. In contrast, the third trimester is characterized by a catabolic phase, with increased levels of free fatty acids, hypertriglyceridemia, and enhanced lipolysis. Physiological insulin resistance during late pregnancy facilitates nutrient transfer to the fetus [89]. These adaptations lead to enhanced adipocyte lipolysis, elevated circulating triglyceride (TG) levels, and increased concentrations of triglyceride-rich lipoproteins, including very low-density lipoproteins (VLDL) [90]. In pregnancies complicated by GDM, these adaptive mechanisms become dysregulated. Women with GDM exhibit significantly higher triglyceride levels and reduced HDL cholesterol concentrations, particularly during the second and third trimesters [91]. Huhtala et al. reported increased levels of VLDL triglycerides, phospholipids, and total triglycerides in lipoproteins from pregnant women with GDM, with VLDL levels positively associated with insulin resistance [92]. Increased maternal lipolysis and altered lipid metabolism result in elevated circulating free fatty acids and triglyceride-rich lipoproteins, which may impair placental function and enhance maternal–fetal lipid transfer [93]. Lipidomic analyses have demonstrated that GDM is associated with increased plasma concentrations of specific triglycerides and diglycerides containing saturated and monounsaturated fatty acids [94].

These alterations have been linked to adverse pregnancy outcomes, including fetal macrosomia, preeclampsia, preterm birth, and fetal distress [95]. A higher incidence of postpartum glucose intolerance was associated with changes in LDL cholesterol in pregnant women with GDM, according to a cohort study [96]. In parallel, disturbances in amino acid metabolism have been consistently observed in GDM. Increased plasma levels of branched-chain amino acids (BCAAs), aromatic amino acids, and gluconeogenic amino acids have been associated with insulin resistance and an increased risk of developing GDM early in pregnancy [97,98,99,100].

Dysregulated amino acid availability may alter nutrient-sensing pathways such as mTOR and JNK, impairing β-cell function and promoting glucose intolerance [101]. Altered concentrations of amino acids in maternal blood, cord blood, and amniotic fluid further support the presence of dysregulated placental transport in GDM, leading to in-creased substrate availability for fetal adipose tissue development and lipogenesis [102]. These metabolic disturbances resemble alterations observed in T2DM mellitus and may contribute to long-term metabolic risk in affected women [103]. Oxidative stress is closely associated with altered amino acid metabolism.

Elevated amounts of aromatic amino acids (AAAs) and BCAAs have been associated with oxidative damage and increased ROS production. Specifically, elevated serum levels of cysteine, phenylalanine, and tyrosine were observed in 80 insulin-treated patients with T2DM, together with markers of increased oxidative stress [104]. Furthermore, the production of glutathione, a key intracellular antioxidant, and consequently the cell’s ability to neutralize ROS are influenced by the availability of amino acids, including glutamine, cysteine, and glycine [105]. Collectively, dysregulation of lipid and amino acid metabolism in GDM contributes to placental metabolic overload, creating a cellular environment that predisposes to mitochondrial dysfunction and the activation of oxidative and inflammatory stress pathways.

### 4.4. Oxidative Stress and Inflammation

Oxidative stress and inflammation in GDM should be considered downstream consequences of placental metabolic overload rather than primary initiating factors. Chronic hyperglycemia, hyperinsulinemia, and dyslipidemia activate oxidative pathways, including the polyol pathway, anaerobic glycolysis, and the mitochondrial respiratory chain, resulting in excessive production of ROS and reactive nitrogen species (RNS) [106,107,108,109,110].

Elevated ROS levels trigger inflammatory signaling cascades regulated by NF-κB, MAPK, and JNK, leading to increased expression of pro-inflammatory cytokines and chemokines such as TNF-α, IL-6, MCP-1, and IL-1β [111,112,113,114]. These mediators further amplify oxidative stress, creating a self-perpetuating cycle of inflammation and cellular damage within the placental tissue.

Oxidative stress directly impairs placental structure and function by altering membrane lipids, reducing membrane fluidity, and disrupting the activity of nutrient transporters, including GLUT1/3, FATP, and SNAT [115,116]. In addition, ROS-induced mitochondrial damage leads to cytochrome c release and activation of apoptotic pathways, ultimately resulting in trophoblast cell death and impaired maternal–fetal oxygen exchange [117].

Hypoxia-related activation of hypoxia-inducible factor-1α (HIF-1α) further suppresses fatty acid β-oxidation and promotes anaerobic glycolysis, exacerbating metabolic inefficiency [118,119,120]. Cellular redox imbalance also influences AMPK and mTOR signaling, linking oxidative stress to altered placental nutrient sensing [121]. Moreover, excessive oxidative stress can induce aberrant epigenetic modifications by altering DNA methyltransferase and histone deacetylase activity. Differential methylation of genes involved in trophoblast proliferation and nutrient transport has been reported in placentas from women with GDM, contributing to adverse fetal programming and increased long-term metabolic risk in the offspring [49,122,123]. These molecular and metabolic alterations provide the mechanistic basis for the identification of circulating and imaging biomarkers aimed at the early detection of GDM. Dysregulation of placental metabolic pathways has been consistently associated with adverse obstetric and neonatal outcomes in GDM. Altered lipid metabolism and maternal hypertriglyceridemia have been linked to increased risk of fetal macrosomia and preeclampsia, even in pregnancies under glycemic control [95]. Similarly, disturbances in amino acid metabolism, particularly involving BCAAs and AAAs, contribute to insulin resistance, enhanced fetal adiposity, and long-term metabolic risk in the offspring [97,98,99,100,102]. Moreover, activation of nutrient-sensing pathways such as mTOR and impairment of AMPK signaling promote excessive placental nutrient transfer, while oxidative stress-induced trophoblast dysfunction has been associated with placental insufficiency, fetal distress, and altered fetal growth trajectories [101,106,117,121].

**Figure 1:**
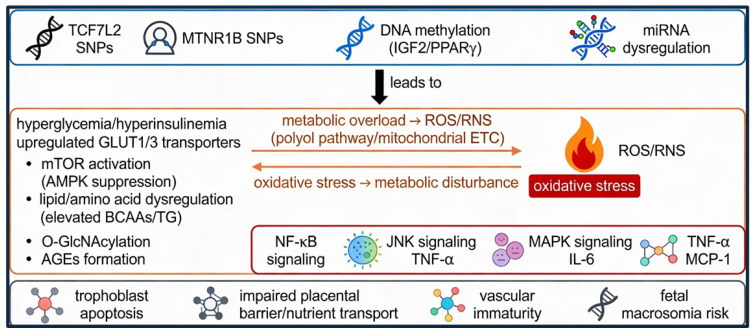
Overview of molecular pathways involved in placental metabolic dysfunction.

The causal chain among genetic, epigenetic factors and metabolic pathway disturbances, and the interaction between oxidative stress and inflammatory pathways are summarized in Figure 1.

Genetic variants in TCF7L2 (transcription factor 7-like 2) and MTNR1B (melatonin receptor 1B), epigenetic modifications including DNA methylation of IGF2 (insulin-like growth factor 2) and PPARγ (peroxisome proliferator-activated receptor gamma), and miRNA dysregulation contribute to maternal hyperglycemia and hyperinsulinemia. These alterations promote metabolic overload and increased production of ROS/RNS (reactive oxygen and nitrogen species) via the polyol pathway and mitochondrial ETC (electron transport chain), leading to oxidative stress and activation of inflammatory signaling pathways (NF-κB, nuclear factor kappa B; JNK, c-Jun N-terminal kinase; MAPK, mitogen-activated protein kinase). The resulting increase in TNF-α (tumor necrosis factor alpha), IL-6 (interleukin-6), and MCP-1 (monocyte chemoattractant protein-1) contributes to trophoblast apoptosis, impaired placental function, vascular immaturity, and increased risk of fetal macrosomia. Color-coded blocks represent distinct but interconnected biological levels: genetic and epigenetic susceptibility (blue), placental metabolic pathway dysregulation and oxidative stress (orange/red), and downstream inflammatory signaling and functional placental outcomes (gray), highlighting the progressive and integrated nature of GDM pathophysiology.

## 5. Non-Invasive Biomarkers for the Early Diagnosis of GDM

The identification of non-invasive biomarkers for GDM is closely linked to the underlying placental metabolic and stress-related pathways described above. Circulating metabolites, lipids, inflammatory mediators, and placental-derived nucleic acids reflect distinct stages of metabolic overload, oxidative stress, and inflammatory activation occurring at the maternal–fetal interface.

### 5.1. Serum Biomarkers

The current gold standard for diagnosing gestational diabetes (GDM) is the oral glucose tolerance test. It is usually performed between the 24th and 28th weeks of gestation. However, many of the pathophysiological mechanisms underlying GDM are already established at the time of diagnosis [124]. Identifying early, non-invasive indicators is essential to diagnose women at highest risk of GDM in the early stages of pregnancy. This could allow dietary and therapeutic approaches to be planned to prevent further problems. The diagnostic efficacy of biomarkers is currently being evaluated on non-invasively retrieved biological substrates (e.g., blood, urine, and maternal stool). The potential use of proteins and metabolites as early indicators for the diagnosis of GDM has been the subject of numerous studies, often with contradictory results.

In first-trimester pregnant women enrolled in their study, Tramontana et al. [125] and Ravnsborg et al. [126] linked higher serum levels of the protein afamin to GDM. Interestingly, in the serum of pregnant women who subsequently developed GDM, sex hormone-binding globulin (SHBG), adiponectin, C-reactive protein (CRP), afamin, serum amyloid P component, and vitronectin were also identified as potential biomarkers [126,127,128]. However, because the same biomarker is often statistically significant in one study but not another, the limited number of cases, variations in analytical methodology, and the timing of blood sampling are the reasons for the conflicting results observed across studies [126,127,129]. Single-stranded non-coding RNAs, or miRNAs, are 20–24 nt long and regulate gene expression at the post-transcriptional level. In both healthy and diseased conditions, some miRNAs can remain in the cell in which they were created, while others, known as circulating miRNAs, can be released into the environment [130]. Specific miRNAs extracted from peripheral maternal blood circulation have been associated with pregnancy complications, including preeclampsia [45].

The meta-analysis study by Dinesen and colleagues found that the following biomarkers could be proposed as potential tools for the early prediction of gestational diabetes: miR-29a, miR-330, miR-134, miR-132, miR-16, miR-223, miR-155, miR-122, miR-17, miR-103, miR-125, miR-210, and miR-222 [131]. Li et al. also recommended adding miR-19a-3p, miR-19b-3p, and miR-21-3p to the list of miRNAs to support further research and achieve early and reliable detection of gestational diabetes [131]. Circular RNAs (CircRNAs) are non-coding RNAs enclosed in a ring-like structure. They can be loaded into exosomes, small vesicles secreted by cells. Exosome-derived circRNAs have been associated with several pathologies, including metabolic disorders and cancer [132]. Owing to its elevated expression across different stages of GDM, exosome-derived circRNA_0039480 (hsa_circRNA_0039480) has emerged as a promising biomarker for the early identification of GDM [133].

A common component of circulating cell-free DNA (cf-DNA), repetitive DNA sequences can be extracted from biological fluids such as plasma and used as potential biomarkers for various diseases, such as cancer [134] and cardiovascular disease [135]. Wang et al. demonstrated that GDM can be detected early in pregnancy using a deep learning model derived from cf-DNA [136]. A defining characteristic of GDM is epigenetic alteration. An epigenomic association analysis conducted by Linares-Pineda and colleagues on maternal peripheral blood samples from 32 pregnant women (16 with GDM and 16 without GDM) revealed the presence of three CpGs capable of distinguishing between GDM and non-GDM groups [50]. According to recent data, pre-diagnostic differences in microbiome-derived metabolites from early to mid-pregnancy have been associated with an increased risk of GDM [98]. Recent evidence suggests that placental-derived exosomes and extracellular vesicles may represent promising non-invasive biomarkers in GDM. These vesicles carry proteins, lipids, and nucleic acids reflective of placental metabolic and inflammatory status and have been implicated in maternal–fetal communication. Emerging studies indicate that exosome-associated cargo may provide mechanistic insight into placental dysfunction and improve early risk stratification for GDM [137,138].

#### Serum Biomarkers and Practical Clinical Applications

Many of these molecular signals (including proteins, non-coding RNAs, and epigenetic markers) are detectable in maternal serum early in pregnancy, supporting their potential translation into clinically actionable biomarkers for risk stratification, early diagnosis, and disease monitoring in GDM.

Serum Biomarkers:

- 1st Trimester Risk Stratification (High-Risk Women)
  − Afamin + SHBG: ↑Afamin/↓SHBG at 11–13 w identifies high-risk women (AUC 0.70–0.75); triggers earlier OGTT (14–16 w) in BMI > 30 or family history cases [139]
  − Clinical Action: Reduces unnecessary OGTTs in low-risk (NPV 80%); prioritizes intensive lifestyle counseling
- miRNA & cfDNA Panels: Early Prediction
  − miR-29a/miR-223/miR-16 panel (meta-analysis): AUC 0.72–0.82; Sens 85% at 12 w for GDM development [140]
  − 3 CpG cfDNA signature (*n* = 32): AUC 0.85; discriminates GDM vs. non-GDM before hyperglycemia
  − Clinical Action: Personalized screening—low-risk panel → standard 24 w OGTT; high-risk: immediate intervention
- Exosomal circRNA (circ0039480): Progression Monitoring
  − Sens 85%, Spec 78% across GDM stages; tracks response to lifestyle/insulin
  − Clinical Action: Dynamic monitoring in confirmed GDM; predicts insulin need (PPV 75%).

An overview of emerging circulating biomarkers for the prediction and early diagnosis of gestational diabetes mellitus, including their clinical utility and main limitations, is provided in Table 3.

### 5.2. Sonographic Markers

Ultrasound is an inexpensive, feasible, fast, and non-invasive diagnostic technique that does not require exposure to ionizing radiation and is therefore widely used during pregnancy.

Two- (2D) or three-dimensional (3D) gray-scale ultrasound can provide quantitative measurements of placental size and volume, as well as qualitative assessment of placental morphology. The three-dimensional power Doppler (3D-PD) ultrasonographic tool provides a more comprehensive assessment of placental function by evaluating placental vascularity and microvascular perfusion in a defined region of interest (ROI) [141].

3D-PD has proven useful for assessing endometrial receptivity in infertility and for quantifying tumor vascularization in oncology. Furthermore, in obstetric conditions such as preeclampsia, 3D-PD may more accurately reflect early placental malperfusion. Studies have shown that three-dimensional power Doppler-derived uteroplacental vascularization indices (namely vascularization index (VI), flow index (FI), and vascularization flow index (VFI)) are more strongly correlated with histopathological markers of placental insufficiency than conventional Doppler based on single-vessel waveforms [142].

These emerging ultrasound techniques show potential for detecting early placental changes associated with GDM and may represent valuable tools for early risk prediction and management [143].

Starting from the first weeks of pregnancy, several morphological and microphysiological changes induced by GDM can already appear in the placenta, such as changes in placental surface area, volume and microvascular structure [144].

The main placental abnormalities related to GDM that can be assessed by ultrasound technique are larger placental size and increased thickness of the central region of the placenta [144].

The 2D and 3D ultrasound findings indicate that, although no significant differences are observed during the first trimester, increased placental volume in GDM becomes detectable from the second trimester (approximately 21–24 weeks) and progresses with advancing gestation [145,146]. Similarly, elevated placental thickness becomes evident at the sonographic exam by 24–28 weeks and continues to increase until term [147].

In addition, the fetal sex can also be determined during the late first or early mid-trimester 2D ultrasound. Recent studies have demonstrated that placentas can be morphologically or functionally different, depending on fetal sex, with distinct gene, steroid, and protein expression patterns [148]. Specifically, Giannubilo et al. observed that women pregnant with a male fetus had a significantly higher AUC–OGTT, a more pronounced fetal abdominal fat thickness, and a higher probability of requiring insulin therapy. The authors hypothesized that male fetal sex may contribute to increased maternal insulin resistance, potentially mediated by higher maternal testosterone levels [149].

Microscopically, in GDM, terminal villi show features of immaturity, and alterations in villous vascularization, with a greater distance between the intervillous space and fetal capillaries [10,11,150].

In this context, an increased number of capillary branches is observed in terminal villi, a phenomenon known as chorangiosis, characterized by elevated capillary density [151].

Although it was thought that abnormalities in villous architecture in GDM only appeared from the second trimester onwards, a prospective study by Han et al. demonstrated that 3D-PD ultrasound can detect reduced vascular indices (VI) and vascular flow indices (VFI) in placentas with GDM as early as the 12th week of gestation, suggesting that abnormal villous vascular development may precede the clinical diagnosis of hyperglycemia [143].

A few years earlier, Wong et al. had already demonstrated in a prospective case–control study that both VI and VFI were significantly lower in the GDM group both during the first and the second trimester [145].

Currently, most published studies focus on using only ultrasound or clinical parameters to predict GDM, with little research combining both for early and accurate risk assessment.

A 2025 study by Zhu et al. retrospectively investigated 122 pregnancies that underwent ultrasound for nuchal translucency screening between 11 + 0 weeks and 13 + 6 weeks, collecting data on both the clinical aspects of the patient (age, BMI, miscarriage, gravidity, and parity) and on the placental ultrasound (placental thickness and volume, VI, FI, and VFI).

Univariate analysis revealed statistically significant differences in age, BMI, number of abortions, gravidity, placental volume, VI, FI, and VFI between the GDM and NGDM groups between the GDM group and the NGDM group (*p* < 0.05).

However, the model combining clinical data with ultrasound data showed the best predictive ability (area under the curve 0.866), and a nomogram was constructed for this model [152].

This study may inform future research aimed at developing predictive screening models for GDM that integrate individual risk factors with ultrasound-derived placental measurements.

Reported inconsistencies among studies may be explained by several factors, including differences in gestational age at sample collection, maternal metabolic status, analytical methodologies, and the biological compartment analyzed (e.g., plasma, serum, urine, or placental-derived material). Moreover, the dynamic progression of GDM during pregnancy may result in time-dependent changes in biomarker expression, contributing to inter-study variability.

In conclusion, future efforts should aim to integrate clinical predictive models with genetic risk scores, epigenetic profiling, and serum measurements of lipid metabolites and amino acids to enable early pregnancy screening for GDM. It would be advisable to offer these tests concomitantly with other first-trimester examinations (e.g., nuchal translucency ultrasound or preeclampsia risk assessment), thereby enabling the early initiation of targeted therapeutic interventions aimed at mitigating the adverse effects of GDM on maternal, pregnancy, and fetal outcomes. Thus, non-invasive biomarkers should be interpreted not as isolated diagnostic tools, but as molecular readouts of the genetic, epigenetic, and metabolic disturbances driving placental dysfunction in GDM.

#### Potential Integration of Placental Ultrasound Biomarkers into Routine Clinical Practice

Taken together, these data support the potential translational value of placental ultrasound biomarkers, highlighting their possible integration into routine obstetric evaluation for early risk assessment and monitoring of GDM.

Ultrasound Biomarkers:

- 1st Trimester Placental Vascular Assessment
  − 3D-PD indices VI/FI/VFI at 11–13 w: ↓vascularization predicts GDM (AUC 0.75); combined with clinical nomogram → AUC 0.866
  − Clinical Action: Risk re-stratification—abnormal US + clinical risk → early OGTT/metformin consideration

- 2nd/3rd Trimester Surveillance
  - Placental volume/thickness: ↑volume correlates with glycemic control; monitors treatment efficacy
  - Clinical Action: Serial assessment in GDM (20–32 w); predicts macrosomia/placental insufficiency

An overview of sonographic markers for the prediction and early identification of gestational diabetes mellitus is provided in Table 4.

## 6. Conclusions

The strengths of existing research on diabetic placenta can be identified in the genome-wide association studies (GWAS) that have robustly identified shared loci (e.g., TCF7L2, MTNR1B) between GDM and T2DM, with genetic risk scores (GRS) showing high predictive power, supporting overlapping heritability mechanisms [1,50]. Epigenome-wide association studies (EWAS) on placenta and maternal blood reveal specific differentially methylated CpGs (e.g., cg04802986 in LGR6, TRIM67, enriched in insulin/Wnt pathways, replicated in independent cohorts with 80% sensitivity for biomarkers [49]. Multi-omics integrations (methylome + transcriptome) link hypermethylation to metabolic dysfunctions (mTOR/AMPK imbalance), explaining perinatal outcomes like macrosomia. Analysis of the literature necessarily leads to the identification of several current epidemiological, biological, and technical limitations of genetic and epigenetic scores.

### 6.1. Genetic Risk Scores Limitations

- Modest effect sizes and limited discrimination: most single SNPs and combined GRS increase GDM risk only modestly (e.g., 10% risk increase per allele; OR around 1.8 for MTNR1B, which is insufficient to replace clinical risk factors or OGTT for individual-level prediction. GRS often add only small increments in AUC when combined with age, BMI, family history and obstetric history, so their impact on clinical decision-making is limited in real-world settings.
- Population specificity and poor generalizability: many GRS are derived from European or East Asian cohorts, with under-representation of African, Hispanic or mixed-ancestry populations, so performance may degrade when applied across ancestries. Allele frequencies and linkage disequilibrium patterns differ by ethnicity, meaning that a score calibrated in one population can misclassify risk or widen health disparities in another.
- Gene–environment interactions and clinical interpretability: genetic risk often interacts with obesity, physical inactivity and diet (e.g., mitigating effect of Mediterranean diet), so the same GRS does not translate into a fixed absolute risk across lifestyles. There is no consensus on risk thresholds that should trigger specific interventions (e.g., earlier OGTT, insulin counseling), and guidelines do not yet incorporate GRS-based algorithms.

### 6.2. Epigenetic Biomarkers: Biological and Technical Constraints

- Tissue specificity and sampling issues: most robust signals come from the placenta, which is only available at delivery, whereas clinicians need first-trimester or preconception markers; blood-based methylation or miRNA profiles only partially reflect placental epigenetics. Epigenetic patterns differ between tissues (placenta, maternal blood, cord blood, saliva), so a CpG or miRNA validated in one compartment may not be informative in another.
- Temporal variability and stability: GDM is a dynamic process; DNA methylation and circulating miRNAs change across trimesters, so a single timepoint may misclassify risk or miss early windows of pathophysiology. Few longitudinal studies have tested whether epigenetic marks are stable enough, or reversible with lifestyle or medical treatment, to be used as reliable monitoring tools.
- Causality vs. consequence: many epigenetic differences (e.g., global hypermethylation, TRIM67 promoter changes, Wnt/cadherin pathway DMCs) may be downstream of hyperglycemia, dyslipidemia, oxidative stress or inflammation rather than primary causes of disease.

### 6.3. Analytical and Implementation Barriers

- Assay standardization and reproducibility: Different platforms (arrays, bisulfite sequencing, qPCR panels) and pipelines give variable results; cut-offs for “abnormal” methylation or miRNA expression are not standardized across laboratories. Pre-analytical variables (sample type, processing time, storage, gestational age at collection) significantly influence biomarker levels and contribute to inconsistent findings between studies.
- Cost, complexity and turnaround time: High-throughput genotyping and epigenomic assays remain more expensive and slower than standard OGTT and routine biochemistry in most healthcare systems. Bioinformatic analysis and interpretation require specialized expertise and infrastructure that are not widely available in routine obstetric practice.
- Limited linkage to hard clinical outcomes: for many proposed genetic and epigenetic markers, associations are strongest with GDM diagnosis, but links to clinically critical endpoints (macrosomia, preeclampsia, neonatal morbidity, long-term offspring metabolic disease) are inconsistent or weak. Without robust outcome prediction and clear added value over simple clinical models, payers and guideline panels are unlikely to endorse routine use.

At present, GRS and epigenetic biomarkers are better suited as research tools and components of experimental risk models than as standalone screening tests. Their most realistic near-term role is in integrated multivariable algorithms combining clinical factors, standard labs, imaging and selected molecular markers, ideally validated in large, multi-ethnic prospective cohorts before clinical implementation. The clinical application of research in this field could lead to the development of combined GRS+epigenetic risk scores (ERS) for pre-conception/first trimester screening, for personalized risk stratification. In addition, non-invasive biomarkers (cfDNA CpG, exosomal circRNA) from maternal blood could be validated in the clinical setting for dynamic monitoring, or multi-omics algorithms could be used to predict insulin requirements.

Future research developments could converge on multi-ancestral prospective studies with single-cell epigenomics/placental biobanks to establish causality; integrations with artificial intelligence for predictive models of the evolution of gestational diabetes into type 2 diabetes; or new epigenetic therapies, for example, with HDAC inhibitors or miRNA mimics. Finally, new precision medicine tools could emerge, such as clinical platforms for real-time epigenetic risk scores from blood, reducing disparities in access to testing.

## Figures and Tables

**Table 1:** Genetic variants and genetic risk factors associated with Gestational Diabetes Mellitus (GDM).

Author(s)	Study Focus	Main Findings
Lowe Jr. W. L. [1]	Genetic susceptibility to GDM	Variants in TCF7L2, MTNR1B and GCK impair insulin secretion and glucose tolerance during pregnancy
Zhen J. et al. [30]	GWAS in Chinese population	Identified four GDM susceptibility loci (MTNR1B, CDKAL1, SLC30A8, CPO)
Chang S. et al. [31]	TCF7L2 polymorphisms	Significant association between TCF7L2 variants and GDM risk
Rosta K. et al. [32]	MTNR1B polymorphisms	rs10830963 G allele strongly associated with increased GDM risk
Zhang C. et al. [33]	Systematic review of genetic variants	Identified nine SNPs across seven genes associated with GDM
Fang X. et al. [34]	Genetic risk score (GRS)	Combined SNPs improve prediction of GDM risk
Ding M et al. [35]	SNP analysis in two cohorts	Multiple variants contribute cumulatively to GDM susceptibility
Ekelund M et al. [36]	Postpartum diabetes prediction	Genetic risk score predicts future T2DM after GDM
Ramos-Levi A. et al. [37]	Gene–diet interaction	Mediterranean diet attenuates genetic risk of GDM
Penno J. et al. [38]	HTR2B polymorphism	rs17619600 variant associated with higher GDM risk
Liu Y. et al. [39]	Lin28/let-7 genetic variation	Polymorphisms linked to postprandial glucose dysregulation
Ao D. et al. [40]	KCNQ1 polymorphism	rs2237892 associated with elevated glucose levels and GDM
Monod C. et al. [41]	Family history of diabetes	First- and second-degree relatives with T2DM increase GDM risk
Lewandowska M. [42]	Family history and BMI	Parental diabetes increases GDM risk 3–4 fold
Pervjakova N. et al. [43]	Trans-ancestral GWAS	Genetic overlap between GDM and T2DM confirmed
Kawai V. K. et al. [44]	Polygenic risk score	Each additional T2DM risk allele increases GDM risk by ~10%

Legend: GDM = Gestational Diabetes Mellitus; GWAS = Genome-Wide Association Study; SNP = Single Nucleotide Polymorphism; GRS = Genetic Risk Score; T2DM = Type 2 Diabetes Mellitus.

**Table 2:** Epigenetic modifications associated with placental and maternal alterations in Gestational Diabetes Mellitus (GDM).

Author(s)	Epigenetic Mechanism	Main Findings
Giannubilo S. R. et al. [45]	Placental epigenetic regulation	DNA methylation and miRNA modulation affect placental function and fetal growth
Koukoura O. et al. [46]	Placental DNA methylation	Altered imprinting influences fetal metabolic programming
Stolzenbach F. et al. [47]	Gene-specific methylation	Environmental factors modulate placental leptin and insulin receptor methylation
Reichetzeder C. et al. [48]	Global DNA methylation	Higher global placental methylation in GDM vs. controls
Meyrueix L. P. et al. [49]	EWAS	Epigenetic changes specific to GDM, independent of obesity
Linares-Pineda T. M. et al. [50]	EWAS in maternal blood	Identified CpGs distinguishing GDM from non-GDM pregnancies
Lu S. et al. [51]	Integrated methylome/transcriptome	Altered genes enriched in insulin signaling and secretion pathways
Chen F. et al. [52]	Promoter methylation	TRIM67 hypermethylation correlates with OGTT glucose levels
Dalfrà M. G. et al. [53]	Review of epigenetic reprogramming	Maternal hyperglycemia drives placenta-specific epigenetic changes
Linares-Pineda T. M. et al. [54]	Longitudinal epigenetic study	Transgenerational metabolic susceptibility in offspring
Mitra T. et al. [55]	Environmental epigenetics	Diet and endocrine disruptors influence placental methylation
Kadam I et al. [56]	One-carbon metabolism	Maternal methyl donors affect placental DNA methylation
Kong D. et al. [57]	Epigenetic programming	GDM alters placental epigenetic memory, increasing future metabolic risk
Zhang Z. et al. [58]	Epigenetic mechanisms	DNA methylation, miRNAs, and histone modifications alter endothelial and metabolic function
Linares-Pineda T. M. et al. [59]	Epigenetic risk score	DNA methylation marks for T2DM also predict GDM
Wang W. Et al. [60]	Placental methylation and outcomes	Epigenetic changes detected but weak correlation with cord blood biomarkers

Legend: GDM = Gestational Diabetes Mellitus; EWAS = Epigenome-Wide Association Study; CpG = Cytosine–phosphate–guanine site; miRNA = microRNA.

**Table 3:** Clinical implications of biomarkers.

Biomarker	Association with GDM	Clinical Utility	Limitations
Afamin	↑ in 1st trimester GDM	AUC 0.70 0.75	Small studies, conflicting results
SHBG	↓ in early GDM	AUC 0.68	Overlaps with PCOS/obesity
miRNA panel (miR 29a, miR 223, etc.)	Meta-analysis ↑ sensitivity	AUC 0.72 0.82	Platform variability, no standardization
cfDNA methylation (3 CpGs)	Early discrimination (n = 32)	AUC 0.85	Small cohorts, validation needed
Exosomal circRNA (circ0039480)	↑ across GDM stages	Sens 85%, Spec	Single study, assay complexity

Legend: Upward (↑) and downward (↓) arrows indicate an increase or a decrease, respectively, in biomarker levels or activity in pregnancies complicated by GDM compared with normoglycemic controls. SHBG = sex hormone–binding globulin; miRNA = microRNA; cfDNA = cell-free DNA; CpGs = cytosine–phosphate–guanine sites; AUC = area under the curve; Sens = sensitivity; Spec = specificity; PCOS = polycystic ovary syndrome.

**Table 4:** Sonographic markers.

Parameter	Timing	GDM Finding	Predictive Value
Placental Volume/Thickness	2nd/3rd trimester	↑ volume from 21–24 w	AUC 0.65–0.70
3D-PD Indices (VI, FI, VFI)	12 w+	↓ vascularization	AUC 0.75 1st trimester)
Nomogram Model (clinical + US)	11–13 w	Combined AUC 0.866	Promising but retrospective

Legend: Upward (↑) and downward (↓) arrows indicate an increase or a decrease, respectively, in the reported sonographic parameter in pregnancies complicated by GDM compared with normoglycemic controls. VI = vascularization index; FI = flow index; VFI = vascularization flow index; US = ultrasound; AUC = area under the curve.

## Data Availability

No new data were created or analyzed in this study. Data sharing is not applicable to this article.
